# Reply to: The Respiratory Rate-Oxygenation Index predicts post-extubation high-flow nasal cannula therapy failure in intensive care unit patients: a retrospective cohort study

**DOI:** 10.5935/2965-2774.20230366resp-en

**Published:** 2023

**Authors:** Elsa D. Ibáñez-Prada, Cristian C. Serrano-Mayorga, Yuli V. Fuentes, Luis Felipe Reyes

**Affiliations:** 1 Unisabana Center for Translational Science, School of Medicine, Universidad de La Sabana - Chía, Colombia

## TO THE EDITOR,

We want to thank Francesco Alessandri, Pierfrancesco Tozzi, and Antonio Esquinas for their interest in our study.^(1)^ Their constructive feedback is greatly appreciated. We have thoroughly reviewed their concerns and are pleased to provide the following responses.

It is important to acknowledge that there is a lack of a universally agreed-upon Respiratory Rate-Oxygenation (ROX) cutoff point for predicting high-flow nasal cannula (HFNC) therapy failure. While our study did not specifically focus on establishing a cutoff point, our proposed value falls within the range suggested by several other studies. Although certain research manuscripts have used higher values, recent systematic reviews and meta-analyses conducted in 2022 favored a narrower cutoff point. These articles included 1,751 and 1,933 patients^(2,3)^ and employed cutoff points ranging from 4.2 to 5.4. Thus, our suggested cutoff point of 4.88 aligns closely with this narrow range, reinforcing the robustness of our findings. Nevertheless, more studies are required to validate and standardize these values.

Numerous risk factors contribute to postextubation failure among intensive care unit (ICU) patients. Among these risk factors, some, such as the duration of ventilation, unfortunately remain inaccessible since it cannot be determined in our cohort. Even so, our study analyzed an array of severity indices and scores to measure disease severity and patients’ physiological states before extubation. One notable tool utilized in our evaluation is the Tobin score,^(4)^ which showed no significant difference between groups. The Tobin score establishes a ratio between the respiratory rate and the tidal volume to predict successful extubation outcomes for mechanically ventilated patients. Thus, this score measures a patient’s respiratory parameters and indirectly assesses alterations in the respiratory dynamics that should affect the score result. However, we recognize these variables’ significance and the Tobin score’s limitations.^(5)^ On the other hand, although our patients were preconditioned by bridge therapy when there was no sign of respiratory failure or muscle fatigue, they all met the high-risk reintubation criteria defined by an age older than 65 years, smoking, and the presence of chronic obstructive pulmonary disease or another comorbid condition.

Finally, it is essential to highlight that even when there is no clear cutoff for the ROX index, the mean ROX index value presented in our cohort was not in the “indeterminate range” (3.85 - 4.87). The mean ROX index value identified in our cohort was above 9.2, which is the value suggested as the maximum cutoff point by Junhai et al.^(3)^ In addition, our cohort’s rapid shallow breathing index and ROX index values indicated a successful transition between HFNC and conventional oxygen therapy. However, the patients for whom HFNC therapy failed presented reduced ROX index values compared to those in whom the treatment was successful. Thus, we can conclude that; first, the ROX index is a user-friendly metric capable of identifying patients at an elevated risk of experiencing HFNC therapy failure postextubation. Second, further studies are needed to adequately establish this index’s cutoff value. Third, further prospective investigations are imperative to solidify this index’s applicability as a bridge therapy for ICU patients.
